# Changes over 15 years in the contribution of adiposity and smoking to deaths in England and Scotland

**DOI:** 10.1186/s12889-021-10167-3

**Published:** 2021-02-11

**Authors:** Frederick K. Ho, Carlos Celis-Morales, Fanny Petermann-Rocha, Solange Liliana Parra-Soto, James Lewsey, Daniel Mackay, Jill P. Pell

**Affiliations:** 1 Institute of Health and Wellbeing, University of Glasgow, 1 Lilybank Gardens, Glasgow, G12 8RZ UK; 2 Institute of Cardiovascular and Medical Sciences, University of Glasgow, BHF Glasgow Cardiovascular Research Centre, 126 University Place, Glasgow, G12 8TA UK; 3 Center for Exercise Physiology Research (CIFE), University Mayor, Santiago, Chile; 4 Human Performance Lab, Education, Physical Activity and Health Research Unit, University Católica del Maule, Talca, Chile

**Keywords:** Obesity, Adiposity, Smoking, Mortality, Population attributable fraction

## Abstract

**Background:**

For many years smoking has been the major threat to public health in developed countries. However, smoking prevalence has declined over a period when adiposity has increased. The aim of this study was to determine whether adiposity now accounts for more deaths than smoking in the general population as a whole or sub-groups of it.

**Methods:**

This is a comparative risk assessment study using Health Surveys for England and Scottish Health Surveys from 2003 to 2017. Annual prevalence of overweight, obesity, current and former smoking were obtained and combined using population-based weights. Sex-specific risk ratios for all-cause mortality were obtained from the most recently published meta-analyses. Population attributable fractions across yeas were then estimated.

**Findings:**

Overall, deaths attributable to current/former smoking declined from 23.1% (95% CI 20.6–25.8%) in 2003 to 19.4% (95% CI 17.3–21.6%) in 2017, whilst those attributable to adiposity (overweight or obesity) increased from 17.9% (95% CI 17.3–18.4%) in 2003 to 23.1% (95% CI 22.3–23.8%) in 2017 with cross-over occurring in 2013. Cross-over occurred earlier in men (2011) than women (2014). It occurred in 2006 for those aged over 65 years of age and in 2012 for those aged 45–64 years. Below 45 years, smoking remained the larger contributor to mortality.

**Interpretation:**

Adiposity now accounts for more deaths in England and Scotland than smoking among people in middle- and old-age. National strategies to address adiposity should be a public health priority.

**Supplementary Information:**

The online version contains supplementary material available at 10.1186/s12889-021-10167-3.

## Background

For several decades, smoking has been a major target of public health interventions, because it made the largest contribution to avoidable deaths [1–3]. In 2004, the World Health Organization [4] calculated that smoking was the top ranked risk factor for all high-income countries accounting for 18% of all deaths, including from lung cancer and cardiovascular disease [5], in contrast to adiposity, which was ranked third and accounted for 8%, including from cardiovascular disease and diabetes mellitus [6]. In 2005, 109,164 (19%) deaths in the United Kingdom were attributable to smoking [7].

In common with other developed countries, the prevalence of smoking has fallen in the United Kingdom; from 24% in 2005 to 17% in 2017 [8]. Over the same period, the prevalence of obesity has increased from 23 to 29% [9]. Whilst smoking is still the largest contributor to cancers overall – 15% of all new cancers in 2015 were attributed to smoking compared with 6% attributed to adiposity [10] – adiposity now exceeds smoking in terms of its contribution to a number of specific cancers including bowel, kidney, ovary, and liver [11]. Meanwhile, the longstanding decrease in cardiovascular mortality across high-income countries has slowed in recent years, and may even start to increase in North America, with the increasing prevalence of obesity being postulated as a cause [12].

To our knowledge, there are no recent studies that have compared the relative health burden of smoking and adiposity in the UK population as well as in population subgroups. The aim of this study was to determine whether adiposity now exceeds smoking in terms of its contribution to all-cause deaths, either in the population of England and Scotland as a whole, or within specific age, sex and socioeconomic sub-groups of it.

## Methods

### Prevalence of adiposity and smoking

Annual prevalence of overweight, obesity, current and former smoking were obtained from the Health Surveys for England (HSE) and Scottish Health Surveys (SHeS). These household surveys were conducted to monitor the health of the general population in England and Scotland. The surveys used two-stage stratified random sampling. A random sample of area-based primary sampling units (based on postcode sectors) were drawn, from which a random sample of postal addresses were selected. All adults (≥16 years of age) and up to 4 children (< 16 years of age) in the selected households were invited to participate the health surveys. Household response rates ranged from 58 to 73% and individual response rates ranged from 54 to 66% with a general declining trend over 2003 to 2017. People who lived in institutions were excluded from the surveys. The HSE was conducted every year from 2003 to 2017. The SHeS was conducted in 2003, and then annually from 2008 to 2017. The prevalence in Scotland in the missing years were interpolated using cubic splines [13] fitted on the available Scottish prevalence data.

Data were collected via face-to-face interviews (e.g. sociodemographic information and smoking status). Physical measurements (e.g. body height and weight) were conducted by survey administrators in all years for HES and since 2012 for SHeS. Prior to that, physical measurements in SHeS were conducted in a follow-up visit by nurses. Smoking status was self-reported using the same question prompt across years and was categorised into never, former and current smoker. Height, to the nearest 1 mm, and weight, to the nearest 0.1 kg, were measured by trained interviewers or nurses using standardised stadiometers and scales. Body mass index (BMI) was calculated using the formula (weight in kg) / (height in m)^2^, and was used to classify participants into: non-overweight or obese (<25 kg/m^2^), overweight (25 to <30 kg/m^2^), class I obese (30 to <35 kg/m^2^), class II obese (35 to <40 kg/m^2^) and class III obese (≥40 kg/m^2^).

In this study, participants aged under 16 years were excluded because there are no reliable risk ratio estimates. Only population representative samples from the surveys were included and boost samples targeting a subset of the population were excluded. Survey weightings were used to estimate the population prevalence in England and Scotland separately. These were combined, using weightings based on the annual population estimates in the two countries, to produce annual prevalence for England and Scotland combined [14].

### Risk ratios of adiposity and smoking

Risk ratios (RRs) were used for the estimation of population attributable fraction. RR is the ratio of absolute risk between exposed (e.g. smoker, BMI > =30) and reference (e.g. never smoker, BMI < 25) groups, and is a measure of relative risk. A systematic literature search was conducted, using PubMed, to determine the RRs of dying from overweight, obese, current and former smoking using these keywords: (‘all-cause mortality’ OR ‘total mortality’) AND ((‘smoking’ OR ‘smoke’ OR ‘cigarette’) OR (‘overweight’ OR ‘obesity’ OR ‘BMI’ OR ‘body mass index’ OR ‘excess weight’)) AND ‘meta-analysis’ AND ‘prospective’. The RRs, and confidence intervals (CIs), reported in the most recent meta-analysis were extracted for this study because they cover the greatest number of studies and overlap with the study period. Because sex is commonly an effect modifier, sex-specific RRs were used. This meta-analysis for smoking included 17 studies; all studies adjusted for age and sex, and 9 also adjusted for blood pressure, 9 for alcohol consumption, and 6 for physical activity. The pooled RRs (95% CIs) applied to current smoking in women and men were 1.80 (1.59–2.04) and 1.90 (1.72–2.10) respectively and to former smoking were 1.32 (1.23–1.40) and 1.34 (1.27–1.40) respectively [15]. The meta-analysis for adiposity included 157 and 141 studies for men and women respectively and reported age and sex adjusted hazard ratios (HRs) among never-smokers without pre-existing chronic conditions [16]. For women, the pooled HRs (95% CI) for overweight, obese I, II and III were: 1.08 (1.07–1.10), 1.37 (1.34–1.40), 1.86 (1.79–1.93), and 2.73 (2.57–2.91). The respective values for men were: 1.12 (1.11–1.13), 1.70 (1.63–1.77), 2.68 (2.54–2.83), and 4.24 (3.77–4.76) [16]. HRs are the ratios of instantaneous incidence rate and were converted into RRs using the formula described elsewhere [17].

### Statistical analyses

The population attributable fraction (PAF) for each risk factor in each year was estimated using the formula $PAF=\frac{\sum {p}_i\times {RR}_i-1}{\sum {p}_i\times {RR}_i}$, where *p*_*i*_ was the prevalence and *RR*_*i*_ was the risk ratios of the i^th^ category of the risk factor [18]. PAF indicates the proportions of deaths in the population that were attributable to the risk factor. People who never smoke and people with BMI <25 kg/m^2^ were the reference groups for smoking and adiposity respectively. The risk attributed by underweight (BMI <18.5 kg/m^2^) was omitted in this study. The confidence interval for the PAF was calculated using 1000 bootstrap repetitions based on the variances of both prevalence and RRs [18]. Bootstrapping using 2000 repetitions resulted in very similar results. The PAFs for each year were then smoothed and plotted using local polynomial regression [19]. Local polynomial regression is a nonparametric technique to smooth a trend line.

Because the RRs for smoking were extracted from a meta-analysis of older adults and might not be applicable to younger people, we also estimated the HRs of former and current smoking using UK Biobank data. UK Biobank is a prospective cohort study of over 500,000 people in England, Scotland and Wales. Death was ascertained using linked data up to May 2020 with a median follow-up of 12 years. A Cox proportional hazard model was used to estimate the HRs of former and current smoking by sex adjusting for age, sex, ethnicity, deprivation, physical activity, and diet. These HRs were then converted into RRs and PAFs were estimated. All analyses were conducted using *R statistical software* version 3.5.3 and the packages *akima* and *pifpaf*.

## Results

The study population comprised 192,239 participants: 136,171 from the HSE and 56,068 from the SHeS (Table 1). Women accounted for 56% of participants and the mean (SD) age was 49.9 (18.6) years. More than two-thirds of participants continued full-time education beyond 16 years of age. The mean (SD) BMI was 27.4 (5.3) kg/m^2^, and around half of the participants were current or former smokers.

**Table 1:** Study participant characteristics

	England *n* = 136,171	Scotland *n* = 56,068
	N (%)	N (%)
**Year**		
2003–2007	50,194 (36.9)	8148 (14.5)
2008–2012	45,063 (33.1)	30,149 (53.8)
2013–2017	40,914 (30.0)	17,771 (31.7)
**Sex**		
Female	75,591 (55.5)	31,462 (56.1)
Male	60,580 (44.5)	24,606 (43.9)
**Age at assessment (years)**		
16–44	55,749 (40.9)	20,615 (36.8)
45–64	45,673 (33.5)	19,847 (35.4)
≥ 65	34,749 (25.5)	15,606 (27.8)
**Age at leaving full-time education (years)**		
< 16	41,818 (30.8)	18,574 (33.2)
≥ 16	93,918 (69.2)	37,295 (66.8)
**Body mass index (kg/m**^**2**^**)**		
< 18.5	1793 (1.5)	683 (1.5)
18.5–< 25.0	40,741 (34.8)	14,809 (31.6)
25.0–< 27.5	24,461 (20.9)	9626 (20.5)
27.5–< 30.0	19,631 (16.8)	8144 (17.4)
30.0–< 35.0	20,603 (17.6)	9208 (19.6)
35.0–< 40.0	6913 (5.9)	3193 (6.8)
≥ 40.0	2791 (2.4)	1232 (2.6)
**Smoking**		
Current	28,722 (21.1)	13,375 (23.9)
Former	35,928 (26.4)	14,094 (25.1)
Never	71,521 (52.5)	28,599 (51.0)

The annual prevalence of overweight, obesity, current and former smoking is consistent with previous reports in England and Scotland (Supplementary Table 1). The percentage of the population with obesity (including Classes I to III) increased steadily from 22.8% in 2003 to 29.1% in 2017. The trend occurred across all age, sex and education sub-groups, but the greatest increases were observed among participants aged 45–64 years (from 27.6 to 35.4%), women (from 23.6 to 30.3%), and those who continued full-time education beyond 16 years of age (from 21.1 to 28.9%). Over the same period, the prevalence of current smoker decreased from 26.2% in 2003 to 18.3% in 2017. The largest decreases were observed among participants aged 16–44 years (from 32.9 to 22.9%), women (from 24.9 to 16.4%), and those who continued full-time education beyond 16 years of age (from 26.2 to 17.3%).

**Fig. 1:**
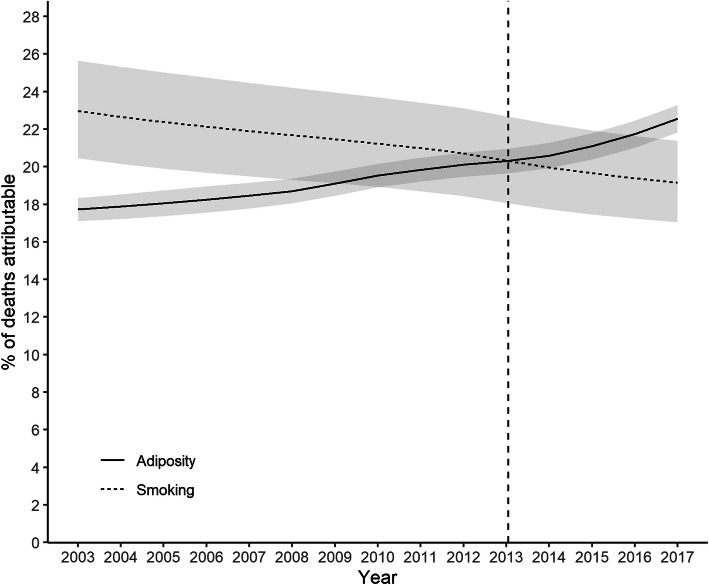
Percentage of all-cause deaths attributable to adiposity and smoking. Shaded areas are 95% confidence bands. Vertical dashed line indicates cross-over

The percentage of all deaths attributable to current or former smoking declined from 23.1% (95% CI 20.6–25.8%) in 2003 to 19.4% (95% CI 17.3–21.6%) in 2017, whilst those attributable to adiposity (overweight or obesity) increased from 17.9% (95% CI 17.3–18.4%) in 2003 to 23.1% (95% CI 22.3–23.8%) in 2017 (Fig. 1). The contribution of adiposity exceeded that of smoking in 2013. In 2017, adiposity contributed to 3.7% more deaths than smoking with non-overlapping confidence intervals.

**Fig. 2:**
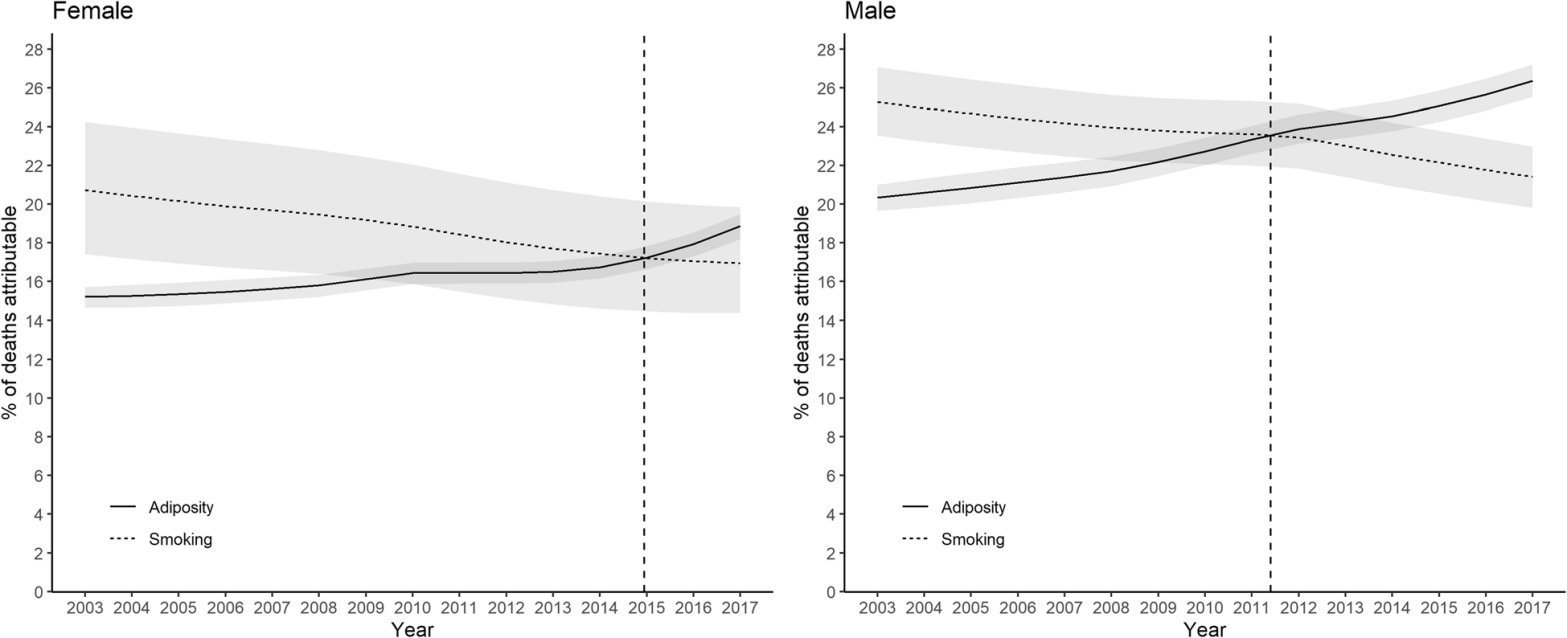
Percentage of all-cause deaths attributable to adiposity and smoking by sex. Shaded areas are 95% confidence bands. Vertical dashed line indicates cross-over

**Fig. 3:**
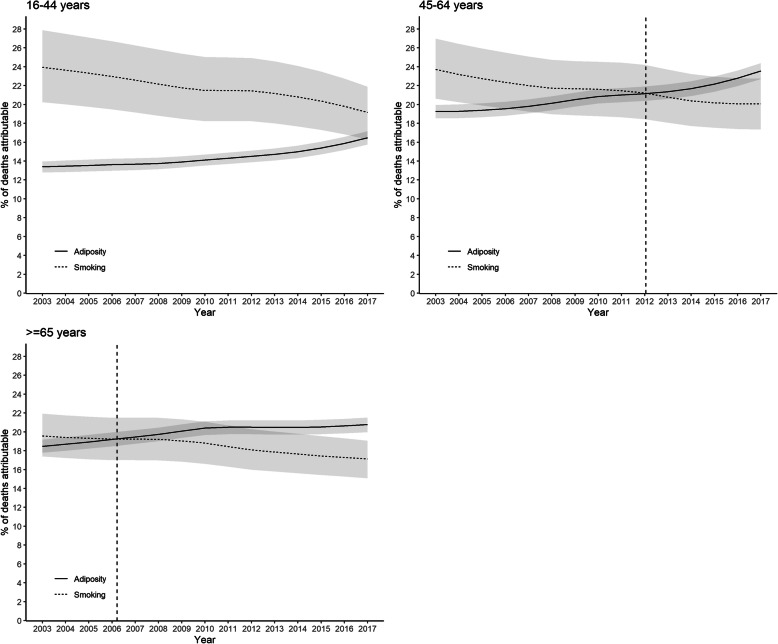
Percentage of all-cause deaths attributable to adiposity and smoking by age group. Shaded areas are 95% confidence bands. Vertical dashed line indicates cross-over

**Fig. 4:**
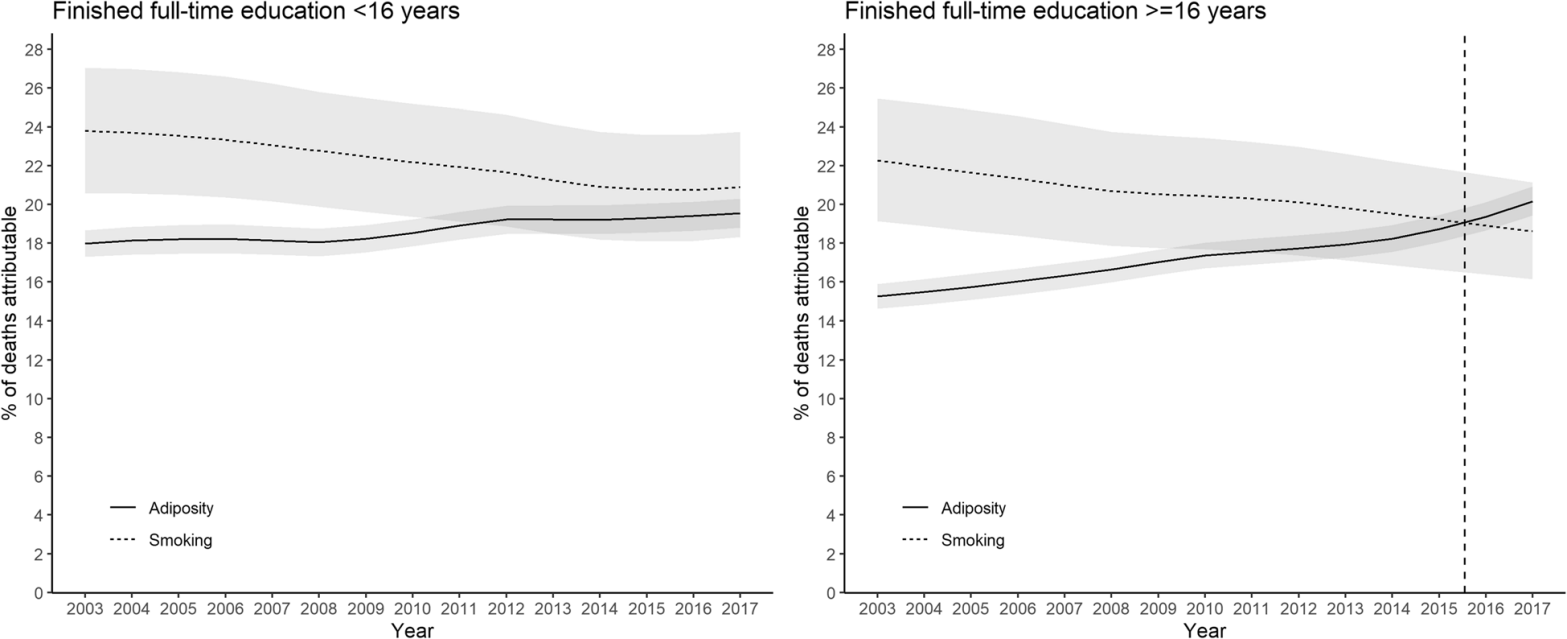
Percentage of all-cause deaths attributable to adiposity and smoking by age at completion of full-time education. Shaded areas are 95% confidence bands. Vertical dashed line indicates cross-over

The general patterns were similar in men and women, but the crossover date occurred earlier in men (2011) than women (2014) (Fig. 2). In 2017, adiposity accounted for 5.2% more deaths in men than smoking with non-overlapping confidence intervals. In women, the difference was 2.2% with overlapping confidence intervals. The temporal trends were more striking in older age-groups. The crossover occurred in 2006 among participants ≥65 years of age compared with 2012 in those aged 45–64 years (Fig. 3). In 2017, adiposity accounted for 3.5% more deaths over 65 years of age than smoking with non-overlapping confidence intervals. Among participants below 45 years of age, smoking remained the larger contributor to deaths over the whole study period. However, the gap decreased dramatically. In 2003 smoking accounted for 10.7% more deaths than adiposity but, by 2017, this had fallen to 2.4% with overlapping confidence intervals. The trend was more pronounced among those who left school at or after 16 years of age than those before (Fig. 4). Unsmoothed estimates of PAFs are shown in Supplementary Tables 2, 3, 4 and 5.

The HRs of former and current smoking among female participants in UK Biobank were estimated to be 1.16 (95% CI 1.08–1.24) and 2.27 (95% CI 2.06–2.49) respectively. Those among male were 1.24 (95% CI 1.18–1.31) and 2.19 (95% CI 2.05–2.34) respectively. The PAFs estimated using these are shown in Supplementary Figs. 1, 2, 3 and 4. The trends were the same as in the main analyses with slightly delayed cross-overs. For example, the contribution of adiposity to mortality exceeded that of smoking in 2014, compared with 2013 in the main analysis.

## Discussion

### Principal findings

This study showed a steady increase in the percentage of deaths attributable to adiposity in England and Scotland over 15 years, and a steady decline in those attributable to smoking over the same period. As a result, adiposity has exceeded smoking as a contributor to deaths since 2014, and the difference has widened.

Whilst the overall trends were fairly consistent across different sub-groups of the population, there were some interesting differences in the timing and magnitude of change. Among older age-groups, the crossover between smoking and adiposity occurred earlier and, therefore, the difference in magnitude is now greater. The earlier crossover in this sub-group reflects their lower prevalence of smoking and higher prevalence of overweight or obese. The trend was also more pronounced in men than women, in spite of men having a higher annual prevalence of smoking than women, and a lower prevalence of obesity.^8 9^ This is largely due to obesity being associated with a higher mortality risk ratio in men than women [16] as well as more overweight men than women; therefore the same increase in prevalence will produce a greater impact on men.

### Strengths and weaknesses of the study

In this study, the internal and external validity of the PAF estimates were enhanced by the use of general population representative data from England and Scotland, and up-to-date risk ratio estimates obtained from meta-analyses of prospective cohort studies. The response rate of both surveys has reduced over time. Any systematic error is likely to take the form of an increasing healthy volunteer effect. Therefore, the estimated PAFs may be underestimates. However, systematic differences in response on the basis of smoking versus obesity are unlikely. Because cohort studies are observational, causation cannot be assumed, and the estimates of risk may be affected by residual confounding. The RRs we extracted for smoking were adjusted for age and sex, and partially for blood pressure, physical activity, and alcohol consumption [15]; while those for adiposity were adjusted for age and sex among never-smokers without pre-existing chronic diseases [16]. Because the effects of lifestyle factors on mortality are mediated through chronic illnesses, the susceptibility to confounding bias between the two sets of RRs should be similar and therefore comparison between the PAFs should be valid. The risk ratio for smoking was based on a 2012 meta-analysis of smokers aged ≥60 years, which may impact its generalisability to our study population as RRs might be different for younger people. Nonetheless, our sensitivity analysis using UK Biobank data provided consistent conclusions. RRs were assumed to remain constant over time and applicable to the UK population even though they were based on studies from various countries. The data from the HSE and SHeS have shown that the median number of cigarettes per day among current smokers has declined from 14.3 and 15.0 in 2003 to 10.0 and 11.3 in 2017 in England and Scotland respectively. This indicates that if the RR of smoking did change during the study, it would likely to be slightly reduced, resulting in our study overestimating the numbers of deaths attributable to smoking in the recent years. The effect sizes of smoking between our selected meta-analysis and studies from UK and Europe were very similar [20], and those of adiposity were slightly stronger in European studies [16]. These indicates that our findings are likely to be a slight underestimate of the increasing contribution of obesity. Smoking deception can result in some current smokers misclassifying themselves as former smokers but this is less common in general population cohorts than smoking-related disease cohorts [21]. BMI is an imperfect measure of adiposity; especially in younger men with high lean body mass. These limitations in the measurement of exposures apply to all study years; therefore, temporal bias is unlikely. Our study was confined to all-cause mortality and the findings will not necessarily apply to other disease-specific outcomes where the relative contribution of the two risk factors may be different.

### Strengths and weaknesses in relation to other studies

Previous studies often reported PAFs as a single point estimates because of the difficulty in combining variances in RRs and prevalence.^7 10^ In our study, we calculated not only PAFs but also the associated confidence intervals, which helps in the comparison of PAFs between sub-groups or across years. For example, the difference in PAFs between adiposity and smoking was only 3.7% in 2017 but the confidence intervals did not overlap giving us greater certainly that the contribution of adiposity was greater than that of smoking in that year. The confidence intervals were wider for smoking than adiposity due to the less precise estimates of RRs [15].

Comparing PAF estimates between studies is problematic because prevalence differs between populations and over time. Nonetheless, our estimates for smoking are not dissimilar to a previous study, which reported that 19% of all deaths in 2005 were attributable to smoking [7]. However, our study’s estimates of the PAF due to adiposity were higher than previous estimates of 2 to 12% [22]. The meta-analysis from which we extracted the RR for adiposity reported the mortality PAF for Europe to be 13.5% [16], based on a prevalence of 33% for overweight and 20% for obesity. In our study the 2017 prevalence of obesity in England and Scotland was much higher at 30.3%. Differences in methodology may also have contributed. For example, in that meta-analysis obesity was treated as a single category whereas, in practice, the RR increases exponentially across obesity classes I, II and III.

The Global Burden of Disease study ranked the top risk factors in the United Kingdom based on disability adjusted life years (DALYs) [23]. It reported that tobacco use was still the top contributor to DALYs in 2017 even though its contribution had fallen by 9.2% since 2007. Over the same period, adiposity rose from fourth to third rank, and its contribution increased by 7.6%. The temporal trends are consistent with our findings. The failure of adiposity to overtake smoking is likely to reflect a greater impact on disability from smoking than adiposity; possibly due to its association with chronic diseases, such as chronic obstructive pulmonary disease (COPD), that impair functioning and wellbeing for a sustained period of time.

### Meaning of the study

Historical efforts to protect the public from the harms of smoking have been successful. Adiposity now contributes to more deaths than smoking, which highlights the need to prioritise strategies to address it, including upstream policies and legislation [24], as well as downstream individual interventions. Middle and older age groups and men, in particular, require support in helping them to reduce their weight to a healthy level.

### Unanswered questions and future research

The current study included the risk of only active and former smoking. Given that vaping and e-cigarette use are growing among long-term former smokers [25], and passive smoking also contributes to mortality [26], future studies should consider whether these specific legacies of smoking require ongoing focus. Adiposity did not outrank smoking among those under 45 years of age and those finished school early when the surveys were undertaken. However, birth cohort studies should be undertaken in the future to fully understand changing risk over time. The association of adiposity with mortality may differ by ethnicity and this might need to be accounted for in future analysis. The combined associations of smoking and adiposity should be considered in future individual-level studies, as there may be interactions between them.

## Conclusions

Since 2014, adiposity has contributed to more deaths in England and Scotland than smoking. Prioritising smoking has been successful at reducing its risk. Interventions to reduce adiposity need to attract the same level of priority among policy makers, practitioners and public health physicians.

## Supplementary Information

**Additional file 1.**

## Data Availability

The HES and SHeS data can be requested from the UK Data Service (https://ukdataservice.ac.uk/). UK Biobank data can be requested from its official portal (https://www.ukbiobank.ac.uk/).

## Abbreviations

PAF: Population attributable fraction
RR: Risk ratio
HSE: Health Survey for England
SHeS: Scottish Health Survey
