# Supplementary material for: Changes over 15 years in the contribution of adiposity and smoking to deaths in England and Scotland
Source: BMC Public Health. 2021 Feb 11;21:169. doi: 10.1186/s12889-021-10167-3 (PMC7876822; doi:10.1186/s12889-021-10167-3)
Supplement: Supplementary file 1 — Additional file 1. [file 12889_2021_10167_MOESM1_ESM.docx]

**Changes over 15 years in the contribution of adiposity and smoking to deaths in England and Scotland**

Supplementary Table 1. Prevalence of adiposity and smoking

| Year | Group | Not  overweight nor obese | Over-weight | Obesity Class I | Obesity Class II | Obesity Class III | Non-smoker | Former smoker | Current smoker |
| --- | --- | --- | --- | --- | --- | --- | --- | --- | --- |
| 2003 | All | 39.2 | 37.9 | 16.4 | 4.6 | 1.8 | 50.1 | 23.7 | 26.2 |
| 2004 | All | 37.9 | 39 | 16.8 | 4.8 | 1.5 | 50.8 | 24.8 | 24.4 |
| 2005 | All | 39.1 | 37.5 | 16.2 | 5.4 | 1.8 | 50.3 | 23.5 | 26.2 |
| 2006 | All | 38.2 | 37.6 | 16.9 | 5.4 | 1.8 | 52.5 | 24 | 23.5 |
| 2007 | All | 38.9 | 37 | 17.3 | 5.4 | 1.5 | 52.3 | 24.2 | 23.5 |
| 2008 | All | 38.2 | 37.1 | 17.2 | 5.7 | 1.8 | 52.9 | 24.3 | 22.8 |
| 2009 | All | 38.5 | 38.2 | 15.7 | 5.5 | 2.1 | 52.9 | 24 | 23.1 |
| 2010 | All | 37 | 36.8 | 18.5 | 5.6 | 2.2 | 54 | 24.8 | 21.2 |
| 2011 | All | 37.9 | 36.9 | 16.5 | 6.1 | 2.5 | 53.4 | 24.6 | 22 |
| 2012 | All | 37.9 | 37.2 | 16.9 | 5.7 | 2.5 | 55.3 | 23.4 | 21.3 |
| 2013 | All | 37.6 | 37.2 | 17 | 5.4 | 2.8 | 53.6 | 25 | 21.4 |
| 2014 | All | 37.8 | 36.2 | 17.5 | 5.8 | 2.7 | 56.5 | 23.5 | 19.9 |
| 2015 | All | 37 | 36 | 18.2 | 6 | 2.9 | 56.1 | 25.2 | 18.8 |
| 2016 | All | 38.1 | 35.2 | 17.1 | 6.6 | 3 | 56.6 | 24.7 | 18.7 |
| 2017 | All | 35.4 | 35.6 | 18.7 | 6.8 | 3.6 | 56.6 | 25.1 | 18.3 |
| 2003 | Aged 16-44 years | 49.5 | 32.8 | 12.4 | 3.6 | 1.6 | 54.8 | 12.3 | 32.9 |
| 2004 | Aged 16-44 years | 48 | 34.7 | 12.5 | 3.8 | 1.1 | 55.7 | 13.8 | 30.6 |
| 2005 | Aged 16-44 years | 49.1 | 32.5 | 12.7 | 4.2 | 1.5 | 53.5 | 13.5 | 33 |
| 2006 | Aged 16-44 years | 48.7 | 33 | 12.8 | 4.1 | 1.5 | 56.9 | 13.9 | 29.2 |
| 2007 | Aged 16-44 years | 49.9 | 32.6 | 12.8 | 3.5 | 1.2 | 56.8 | 13.7 | 29.5 |
| 2008 | Aged 16-44 years | 49.1 | 32.6 | 12.5 | 4.4 | 1.5 | 56.8 | 13.9 | 29.3 |
| 2009 | Aged 16-44 years | 50.5 | 33 | 10.4 | 4.4 | 1.7 | 58.5 | 14.3 | 27.2 |
| 2010 | Aged 16-44 years | 48.8 | 31.6 | 14.1 | 3.7 | 1.9 | 58.4 | 14.8 | 26.8 |
| 2011 | Aged 16-44 years | 50.3 | 32.1 | 11.2 | 4.3 | 2.1 | 58.3 | 14.4 | 27.3 |
| 2012 | Aged 16-44 years | 49.4 | 32.1 | 12.1 | 4.3 | 2.1 | 59.3 | 15 | 25.7 |
| 2013 | Aged 16-44 years | 48.9 | 31.7 | 12.8 | 4.1 | 2.5 | 57.2 | 16 | 26.7 |
| 2014 | Aged 16-44 years | 50.6 | 30.4 | 12.4 | 4.5 | 2.2 | 60.3 | 14.7 | 25 |
| 2015 | Aged 16-44 years | 47.4 | 30.8 | 14.6 | 5.1 | 2 | 60.7 | 15.1 | 24.2 |
| 2016 | Aged 16-44 years | 49.8 | 30.3 | 12.6 | 4.5 | 2.7 | 61 | 16.2 | 22.8 |
| 2017 | Aged 16-44 years | 45.8 | 31.4 | 14.7 | 4.9 | 3.2 | 61.1 | 17 | 22 |
| 2003 | Aged 45-64 years | 30.3 | 42.1 | 19.6 | 5.7 | 2.3 | 45.9 | 29.6 | 24.5 |
| 2004 | Aged 45-64 years | 28.2 | 42.1 | 21.4 | 6.3 | 2 | 46.4 | 30.2 | 23.4 |
| 2005 | Aged 45-64 years | 30.1 | 41.7 | 19.3 | 6.5 | 2.5 | 47.1 | 27.8 | 25.1 |
| 2006 | Aged 45-64 years | 29.1 | 41.5 | 20 | 7 | 2.4 | 49.1 | 28.2 | 22.7 |
| 2007 | Aged 45-64 years | 29.2 | 39.5 | 21.5 | 7.9 | 1.8 | 49.4 | 28.4 | 22.2 |
| 2008 | Aged 45-64 years | 29.5 | 39.8 | 21.7 | 6.8 | 2.2 | 50.9 | 28.6 | 20.5 |
| 2009 | Aged 45-64 years | 28 | 41.8 | 21.3 | 6.5 | 2.5 | 49.6 | 26.2 | 24.2 |
| 2010 | Aged 45-64 years | 27.2 | 40.3 | 22.5 | 7.6 | 2.4 | 52 | 28.1 | 19.9 |
| 2011 | Aged 45-64 years | 28.1 | 40.3 | 20.1 | 8.3 | 3.2 | 50.5 | 27.4 | 22.1 |
| 2012 | Aged 45-64 years | 27.9 | 41.3 | 20.1 | 7.5 | 3.2 | 52 | 25.9 | 22.1 |
| 2013 | Aged 45-64 years | 28.6 | 41.4 | 20.2 | 6.5 | 3.3 | 51.5 | 27 | 21.5 |
| 2014 | Aged 45-64 years | 28.8 | 39 | 21.1 | 7.3 | 3.7 | 54.9 | 25.7 | 19.4 |
| 2015 | Aged 45-64 years | 27.4 | 39.9 | 21.2 | 7.2 | 4.3 | 53.8 | 28.5 | 17.6 |
| 2016 | Aged 45-64 years | 28.5 | 38.3 | 20.7 | 8.6 | 3.9 | 53.1 | 27.4 | 19.5 |
| 2017 | Aged 45-64 years | 27 | 37.5 | 22 | 8.3 | 5.1 | 53.6 | 27.1 | 19.3 |
| 2003 | Aged ≥65 years | 28.1 | 43.8 | 21.3 | 5.1 | 1.7 | 45.5 | 41.8 | 12.7 |
| 2004 | Aged ≥65 years | 28.6 | 44.9 | 19.8 | 5 | 1.6 | 46.1 | 42.6 | 11.3 |
| 2005 | Aged ≥65 years | 28.8 | 43.1 | 20 | 6.5 | 1.7 | 47.6 | 40.3 | 12.1 |
| 2006 | Aged ≥65 years | 27.5 | 42.5 | 21.8 | 6.3 | 1.9 | 47.6 | 40.7 | 11.7 |
| 2007 | Aged ≥65 years | 28.5 | 43.2 | 20.9 | 5.8 | 1.6 | 46.3 | 42 | 11.8 |
| 2008 | Aged ≥65 years | 26.3 | 43.6 | 21.3 | 7 | 1.8 | 47.1 | 41.7 | 11.2 |
| 2009 | Aged ≥65 years | 27 | 44.8 | 19.6 | 6.3 | 2.3 | 45.2 | 43 | 11.8 |
| 2010 | Aged ≥65 years | 25.5 | 42.9 | 22.2 | 7 | 2.4 | 47.2 | 42.1 | 10.7 |
| 2011 | Aged ≥65 years | 26.2 | 42.1 | 22.4 | 6.7 | 2.5 | 47.2 | 42.4 | 10.4 |
| 2012 | Aged ≥65 years | 27.5 | 42 | 22.4 | 5.9 | 2.2 | 51.7 | 38.2 | 10.1 |
| 2013 | Aged ≥65 years | 27.4 | 42.4 | 21.1 | 6.7 | 2.4 | 49.2 | 40.8 | 10 |
| 2014 | Aged ≥65 years | 25.3 | 43.7 | 22.6 | 6.3 | 2.2 | 51.2 | 38.4 | 10.4 |
| 2015 | Aged ≥65 years | 28.7 | 41.3 | 21.4 | 6.1 | 2.5 | 49.5 | 41.9 | 8.6 |
| 2016 | Aged ≥65 years | 26.9 | 41.2 | 21.3 | 8.1 | 2.4 | 52.1 | 39 | 8.9 |
| 2017 | Aged ≥65 years | 25.9 | 41.6 | 22.1 | 8.4 | 2 | 51.6 | 39.4 | 9 |
| 2003 | Female | 43.7 | 32.7 | 15.3 | 5.4 | 2.9 | 55 | 20 | 24.9 |
| 2004 | Female | 42.2 | 34.1 | 15.4 | 5.9 | 2.4 | 54.9 | 21.5 | 23.6 |
| 2005 | Female | 43 | 32.2 | 15.4 | 6.6 | 2.9 | 55.5 | 20.1 | 24.4 |
| 2006 | Female | 43.4 | 31.9 | 15.5 | 6.5 | 2.7 | 56.4 | 21.5 | 22 |
| 2007 | Female | 43 | 32.1 | 16.2 | 6.4 | 2.3 | 56.7 | 21.2 | 22.1 |
| 2008 | Female | 42.5 | 32.3 | 15.9 | 6.6 | 2.8 | 57.1 | 21.9 | 20.9 |
| 2009 | Female | 42.9 | 32.8 | 14.3 | 6.7 | 3.4 | 57.1 | 21.7 | 21.3 |
| 2010 | Female | 41.6 | 31.9 | 16.6 | 6.4 | 3.5 | 58 | 22.3 | 19.7 |
| 2011 | Female | 41.4 | 32.4 | 15.9 | 6.9 | 3.3 | 58.4 | 22 | 19.6 |
| 2012 | Female | 42.4 | 32.2 | 15.9 | 6.3 | 3.2 | 59.6 | 21.3 | 19.1 |
| 2013 | Female | 42.3 | 33.2 | 15.1 | 5.4 | 3.9 | 59 | 22.8 | 18.2 |
| 2014 | Female | 41.4 | 31.4 | 16.9 | 6.7 | 3.6 | 61.2 | 21.2 | 17.6 |
| 2015 | Female | 41.6 | 31.2 | 16.7 | 7 | 3.6 | 59.4 | 23 | 17.6 |
| 2016 | Female | 42.2 | 30.6 | 15.5 | 7.7 | 4 | 61 | 22.5 | 16.5 |
| 2017 | Female | 38.2 | 31.5 | 17.7 | 7.9 | 4.7 | 60.5 | 23.2 | 16.4 |
| 2003 | Male | 34.7 | 43.2 | 17.6 | 3.8 | 0.8 | 45 | 27.6 | 27.5 |
| 2004 | Male | 33.5 | 44.1 | 18.2 | 3.7 | 0.5 | 46.6 | 28.2 | 25.2 |
| 2005 | Male | 35 | 42.9 | 17.1 | 4.2 | 0.8 | 44.7 | 27.2 | 28.1 |
| 2006 | Male | 32.9 | 43.5 | 18.3 | 4.3 | 1 | 48.3 | 26.5 | 25.1 |
| 2007 | Male | 34.7 | 41.8 | 18.3 | 4.4 | 0.7 | 47.6 | 27.3 | 25.1 |
| 2008 | Male | 33.9 | 42 | 18.6 | 4.7 | 0.7 | 48.5 | 26.7 | 24.7 |
| 2009 | Male | 34.1 | 43.7 | 17.2 | 4.3 | 0.7 | 48.6 | 26.4 | 25 |
| 2010 | Male | 32.4 | 41.6 | 20.3 | 4.8 | 0.8 | 49.8 | 27.4 | 22.9 |
| 2011 | Male | 34.4 | 41.4 | 17.1 | 5.3 | 1.8 | 48.2 | 27.3 | 24.5 |
| 2012 | Male | 33.2 | 42.2 | 17.8 | 5 | 1.7 | 50.8 | 25.6 | 23.5 |
| 2013 | Male | 32.8 | 41.2 | 18.9 | 5.5 | 1.6 | 48 | 27.2 | 24.8 |
| 2014 | Male | 34.1 | 41.1 | 18.1 | 4.9 | 1.8 | 51.7 | 25.9 | 22.4 |
| 2015 | Male | 32.3 | 40.9 | 19.7 | 5 | 2.2 | 52.6 | 27.4 | 20 |
| 2016 | Male | 34 | 39.8 | 18.7 | 5.5 | 2.1 | 51.9 | 27 | 21.1 |
| 2017 | Male | 32.6 | 39.8 | 19.6 | 5.5 | 2.4 | 52.5 | 27.2 | 20.3 |
| 2003 | Ed<16 | 35.8 | 38.1 | 18.7 | 5.2 | 2.2 | 44.1 | 30.2 | 25.7 |
| 2004 | Ed<16 | 34.9 | 38.2 | 19.4 | 5.5 | 2 | 46.1 | 28.8 | 25.1 |
| 2005 | Ed<16 | 35.6 | 36.9 | 18.9 | 6.4 | 2.2 | 46 | 29.1 | 24.9 |
| 2006 | Ed<16 | 36.2 | 35.9 | 18.9 | 6.6 | 2.4 | 47 | 29.1 | 23.9 |
| 2007 | Ed<16 | 37.6 | 36.1 | 18.6 | 6.4 | 1.4 | 45.7 | 29.7 | 24.5 |
| 2008 | Ed<16 | 38.8 | 34.5 | 18.2 | 6.6 | 1.9 | 47.7 | 29.4 | 22.9 |
| 2009 | Ed<16 | 35.7 | 39.3 | 17.2 | 5.4 | 2.3 | 47 | 29.8 | 23.3 |
| 2010 | Ed<16 | 37.6 | 34.2 | 20 | 6.2 | 2.1 | 49.9 | 28.7 | 21.4 |
| 2011 | Ed<16 | 38.5 | 32.8 | 18.6 | 7.4 | 2.7 | 47.8 | 29.9 | 22.3 |
| 2012 | Ed<16 | 39.4 | 33.6 | 18.3 | 6.1 | 2.6 | 51.4 | 25.7 | 23 |
| 2013 | Ed<16 | 38 | 34.5 | 17.1 | 6.7 | 3.6 | 50.1 | 28.5 | 21.4 |
| 2014 | Ed<16 | 38.7 | 34.4 | 17.7 | 6.4 | 2.7 | 53.1 | 27.1 | 19.8 |
| 2015 | Ed<16 | 38.4 | 33.4 | 19.7 | 5.4 | 3.2 | 51.2 | 29.4 | 19.4 |
| 2016 | Ed<16 | 40 | 32.5 | 18.1 | 6.5 | 2.8 | 52.5 | 27.7 | 19.8 |
| 2017 | Ed<16 | 37.3 | 33.7 | 18.5 | 7.5 | 3 | 49.7 | 29.7 | 20.5 |
| 2003 | Ed>=16 | 41.1 | 37.8 | 15.2 | 4.2 | 1.7 | 53.8 | 20 | 26.2 |
| 2004 | Ed>=16 | 39.6 | 39.4 | 15.3 | 4.5 | 1.2 | 53.6 | 22.6 | 23.8 |
| 2005 | Ed>=16 | 40.9 | 37.8 | 14.9 | 4.9 | 1.6 | 52.9 | 20.6 | 26.5 |
| 2006 | Ed>=16 | 39.1 | 38.4 | 16 | 4.9 | 1.6 | 55.3 | 21.7 | 23.1 |
| 2007 | Ed>=16 | 39.4 | 37.3 | 16.7 | 5 | 1.6 | 55.6 | 21.7 | 22.7 |
| 2008 | Ed>=16 | 38 | 38.3 | 16.8 | 5.3 | 1.7 | 55.6 | 22.1 | 22.3 |
| 2009 | Ed>=16 | 39.7 | 37.8 | 15.1 | 5.5 | 2 | 55.8 | 21.5 | 22.7 |
| 2010 | Ed>=16 | 36.7 | 37.9 | 17.8 | 5.4 | 2.2 | 55.8 | 23.2 | 21 |
| 2011 | Ed>=16 | 37.7 | 38.5 | 15.7 | 5.6 | 2.5 | 55.8 | 22.6 | 21.6 |
| 2012 | Ed>=16 | 37.3 | 38.4 | 16.3 | 5.5 | 2.4 | 56.9 | 22.6 | 20.5 |
| 2013 | Ed>=16 | 37.4 | 38.2 | 16.9 | 5 | 2.5 | 55.2 | 23.7 | 21.1 |
| 2014 | Ed>=16 | 37.5 | 36.8 | 17.4 | 5.6 | 2.7 | 58 | 22.4 | 19.6 |
| 2015 | Ed>=16 | 36.6 | 36.8 | 17.7 | 6.2 | 2.8 | 57.7 | 23.9 | 18.3 |
| 2016 | Ed>=16 | 37.5 | 36.1 | 16.8 | 6.6 | 3.1 | 58 | 23.8 | 18.2 |
| 2017 | Ed>=16 | 34.9 | 36.2 | 18.7 | 6.5 | 3.7 | 58.8 | 23.9 | 17.3 |

Not overweight nor obese: BMI<25 kg/m^2^, overweight: BMI 25 to <30 kg/m^2^, class I obesity: BMI 30 to <35 kg/m^2^, class II obesity: BMI 35 to <40 kg/m^2^, and class III obesity: BMI≥40 kg/m^2^. Ed<16: completed full-time education before 16 years of age; Ed>=16: completed full-time education on or after 16 years of age.

Supplementary Table 2. Fraction of deaths attributable to adiposity and smoking

| **Year** | **Adiposity** | **Smoking** |
| --- | --- | --- |
| 2003 | 17.90 (17.33-18.42) | 23.14 (20.60-25.79) |
| 2004 | 17.36 (16.64-18.11) | 22.23 (19.80-24.90) |
| 2005 | 18.30 (17.57-19.01) | 22.86 (20.29-25.60) |
| 2006 | 18.80 (18.17-19.44) | 21.75 (19.33-24.25) |
| 2007 | 18.22 (17.45-19.02) | 21.92 (19.48-24.58) |
| 2008 | 18.65 (18.06-19.23) | 21.67 (19.32-24.19) |
| 2009 | 18.80 (18.12-19.45) | 21.74 (19.39-24.22) |
| 2010 | 19.75 (19.14-20.37) | 20.91 (18.62-23.32) |
| 2011 | 20.14 (19.51-20.74) | 21.10 (18.83-23.52) |
| 2012 | 20.08 (19.43-20.75) | 20.54 (18.23-23.03) |
| 2013 | 20.10 (19.44-20.74) | 20.81 (18.57-23.17) |
| 2014 | 20.34 (19.64-21.01) | 19.67 (17.43-21.97) |
| 2015 | 21.26 (20.57-21.95) | 19.34 (17.17-21.59) |
| 2016 | 20.97 (20.26-21.70) | 19.29 (17.10-21.52) |
| 2017 | 23.05 (22.31-23.77) | 19.35 (17.28-21.63) |

Numbers are percent of deaths (95% CI) attributable to adiposity and smoking.

Supplementary Table 3. Fraction of deaths attributable to adiposity and smoking by sex

|  | **Female** | | **Male** | |
| --- | --- | --- | --- | --- |
| **Year** | **Adiposity** | **Smoking** | **Adiposity** | **Smoking** |
| 2003 | 15.28 (14.78-15.73) | 20.79 (17.47-24.27) | 20.60 (19.97-21.19) | 25.56 (23.81-27.35) |
| 2004 | 14.81 (14.15-15.46) | 20.48 (17.26-24.04) | 19.99 (19.19-20.84) | 24.03 (22.40-25.79) |
| 2005 | 15.98 (15.34-16.64) | 20.06 (16.79-23.66) | 20.69 (19.87-21.45) | 25.75 (23.89-27.59) |
| 2006 | 15.41 (14.90-15.96) | 19.60 (16.50-22.93) | 22.29 (21.53-23.02) | 23.96 (22.25-25.61) |
| 2007 | 15.40 (14.72-16.07) | 19.76 (16.57-23.25) | 21.13 (20.25-22.05) | 24.15 (22.48-25.95) |
| 2008 | 15.63 (15.15-16.12) | 19.47 (16.43-22.84) | 21.75 (21.05-22.42) | 23.93 (22.28-25.58) |
| 2009 | 16.32 (15.68-16.89) | 19.48 (16.47-22.77) | 21.35 (20.63-22.08) | 24.05 (22.39-25.72) |
| 2010 | 16.42 (15.89-16.96) | 18.55 (15.57-21.61) | 23.18 (22.48-23.87) | 23.35 (21.75-25.08) |
| 2011 | 16.77 (16.20-17.27) | 18.50 (15.59-21.63) | 23.62 (22.92-24.31) | 23.77 (22.18-25.47) |
| 2012 | 16.46 (15.90-17.01) | 18.08 (15.07-21.32) | 23.79 (23.06-24.60) | 23.08 (21.49-24.79) |
| 2013 | 15.90 (15.34-16.46) | 17.83 (15.01-20.82) | 24.42 (23.65-25.15) | 23.88 (22.23-25.59) |
| 2014 | 17.02 (16.46-17.58) | 17.06 (14.24-19.96) | 23.76 (22.90-24.55) | 22.35 (20.70-24.04) |
| 2015 | 17.08 (16.48-17.67) | 17.51 (14.78-20.45) | 25.57 (24.78-26.36) | 21.23 (19.63-22.76) |
| 2016 | 17.44 (16.79-18.05) | 16.85 (14.11-19.70) | 24.59 (23.84-25.46) | 21.81 (20.18-23.40) |
| 2017 | 19.23 (18.58-19.84) | 17.02 (14.48-19.97) | 26.99 (26.14-27.82) | 21.75 (20.16-23.34) |

Numbers are percent of deaths (95% CI) attributable to adiposity and smoking.

Supplementary Table 4. Fraction of deaths attributable to adiposity and smoking by age group

|  | **16-44 years** | | **45-64 years** | | **65+ years** | |
| --- | --- | --- | --- | --- | --- | --- |
| **Year** | **Adiposity** | **Smoking** | **Adiposity** | **Smoking** | **Adiposity** | **Smoking** |
| 2003 | 13.47 (12.91-13.99) | 24.21 (20.45-28.23) | 19.23 (18.59-19.91) | 23.48 (20.36-26.72) | 18.75 (18.13-19.45) | 19.84 (17.67-22.26) |
| 2004 | 13.15 (12.44-13.80) | 22.82 (19.39-26.57) | 19.46 (18.63-20.21) | 23.44 (20.58-26.69) | 17.93 (17.23-18.67) | 19.12 (16.93-21.42) |
| 2005 | 13.79 (13.14-14.42) | 24.24 (20.46-28.02) | 19.02 (18.24-19.78) | 23.10 (20.16-26.40) | 19.23 (18.46-20.01) | 19.21 (17.03-21.39) |
| 2006 | 14.09 (13.46-14.68) | 22.50 (19.01-26.08) | 19.75 (19.07-20.46) | 21.93 (19.26-25.01) | 19.87 (19.20-20.63) | 19.08 (16.83-21.35) |
| 2007 | 13.20 (12.54-13.85) | 22.76 (19.55-26.51) | 19.85 (19.03-20.65) | 22.16 (19.48-25.35) | 19.00 (18.24-19.75) | 19.28 (17.05-21.60) |
| 2008 | 14.00 (13.44-14.57) | 22.62 (19.11-26.35) | 20.06 (19.35-20.74) | 21.08 (18.35-23.99) | 19.75 (19.06-20.44) | 18.98 (16.82-21.27) |
| 2009 | 13.64 (13.01-14.28) | 21.46 (18.14-25.02) | 20.26 (19.53-21.04) | 22.73 (19.76-25.78) | 19.71 (18.98-20.41) | 19.42 (17.21-21.73) |
| 2010 | 14.32 (13.76-14.89) | 21.20 (17.93-24.70) | 21.07 (20.34-21.81) | 20.74 (18.07-23.48) | 20.85 (20.09-21.57) | 18.73 (16.49-20.94) |
| 2011 | 14.38 (13.77-14.94) | 21.48 (18.39-25.04) | 21.40 (20.63-22.14) | 21.72 (18.80-24.79) | 20.84 (20.08-21.56) | 18.67 (16.53-20.98) |
| 2012 | 14.32 (13.72-14.94) | 21.39 (18.16-24.79) | 21.01 (20.22-21.77) | 21.24 (18.51-24.23) | 20.22 (19.46-21.00) | 17.56 (15.49-19.72) |
| 2013 | 14.90 (14.25-15.56) | 21.92 (18.62-25.42) | 20.88 (20.15-21.70) | 21.37 (18.62-24.40) | 20.21 (19.49-20.92) | 17.94 (15.92-20.11) |
| 2014 | 14.68 (13.99-15.35) | 20.44 (17.37-23.71) | 21.63 (20.86-22.45) | 19.79 (17.20-22.60) | 20.50 (19.77-21.25) | 17.99 (15.90-20.09) |
| 2015 | 15.69 (15.03-16.36) | 19.81 (16.74-22.97) | 22.52 (21.70-23.29) | 19.50 (16.96-22.07) | 20.29 (19.52-21.04) | 17.51 (15.48-19.73) |
| 2016 | 15.30 (14.64-15.94) | 20.16 (17.19-23.00) | 22.31 (21.51-23.11) | 20.06 (17.41-22.69) | 20.98 (20.18-21.78) | 17.14 (15.23-19.16) |
| 2017 | 16.77 (16.06-17.51) | 19.15 (16.30-21.93) | 23.78 (22.91-24.61) | 20.40 (17.61-23.22) | 20.65 (19.86-21.38) | 17.14 (15.05-19.06) |

Numbers are percent of deaths (95% CI) attributable to adiposity and smoking.

Supplementary Table 5. Fraction of deaths attributable to adiposity and smoking by age finishing continuous education

|  | **<16 years** | | **≥16 years** | |
| --- | --- | --- | --- | --- |
| **Year** | **Adiposity** | **Smoking** | **Adiposity** | **Smoking** |
| 2003 | 17.84 (17.19-18.46) | 23.86 (20.69-27.01) | 15.35 (14.78-15.92) | 22.15 (19.04-25.20) |
| 2004 | 17.89 (17.11-18.66) | 23.49 (20.30-26.84) | 15.29 (14.60-16.02) | 21.76 (18.73-25.06) |
| 2005 | 18.66 (17.92-19.45) | 23.76 (20.68-27.15) | 15.74 (14.98-16.47) | 22.53 (19.35-26.02) |
| 2006 | 19.27 (18.50-19.99) | 23.09 (20.19-26.37) | 16.18 (15.57-16.81) | 21.05 (18.12-24.13) |
| 2007 | 17.49 (16.70-18.25) | 23.74 (20.74-26.99) | 16.44 (15.74-17.14) | 20.61 (17.74-23.71) |
| 2008 | 18.17 (17.49-18.80) | 22.41 (19.65-25.32) | 16.56 (15.91-17.13) | 20.91 (18.18-23.98) |
| 2009 | 17.91 (17.25-18.67) | 22.63 (19.67-25.69) | 16.64 (15.96-17.31) | 20.77 (17.84-23.91) |
| 2010 | 18.45 (17.74-19.13) | 21.61 (18.82-24.36) | 17.75 (17.10-18.44) | 20.12 (17.52-22.99) |
| 2011 | 19.52 (18.83-20.24) | 22.48 (19.65-25.71) | 17.73 (17.08-18.40) | 20.41 (17.61-23.41) |
| 2012 | 18.67 (17.93-19.38) | 21.69 (18.96-24.78) | 17.74 (17.05-18.40) | 19.91 (17.25-22.78) |
| 2013 | 19.79 (18.99-20.50) | 21.40 (18.59-24.11) | 17.60 (16.94-18.31) | 20.31 (17.56-23.19) |
| 2014 | 18.85 (18.13-19.61) | 20.31 (17.51-23.13) | 18.06 (17.39-18.76) | 19.33 (16.59-21.95) |
| 2015 | 19.22 (18.48-19.97) | 20.69 (18.03-23.36) | 18.74 (18.04-19.44) | 18.90 (16.40-21.50) |
| 2016 | 18.62 (17.92-19.35) | 20.45 (17.79-23.24) | 19.26 (18.57-20.01) | 18.93 (16.46-21.53) |
| 2017 | 20.14 (19.34-20.87) | 21.24 (18.68-24.19) | 20.29 (19.54-21.07) | 18.75 (16.21-21.30) |

Numbers are percent of deaths (95% CI) attributable to adiposity and smoking.

Supplementary Figure 1. Percentage of all-cause deaths attributable to adiposity and smoking using alternative RRs


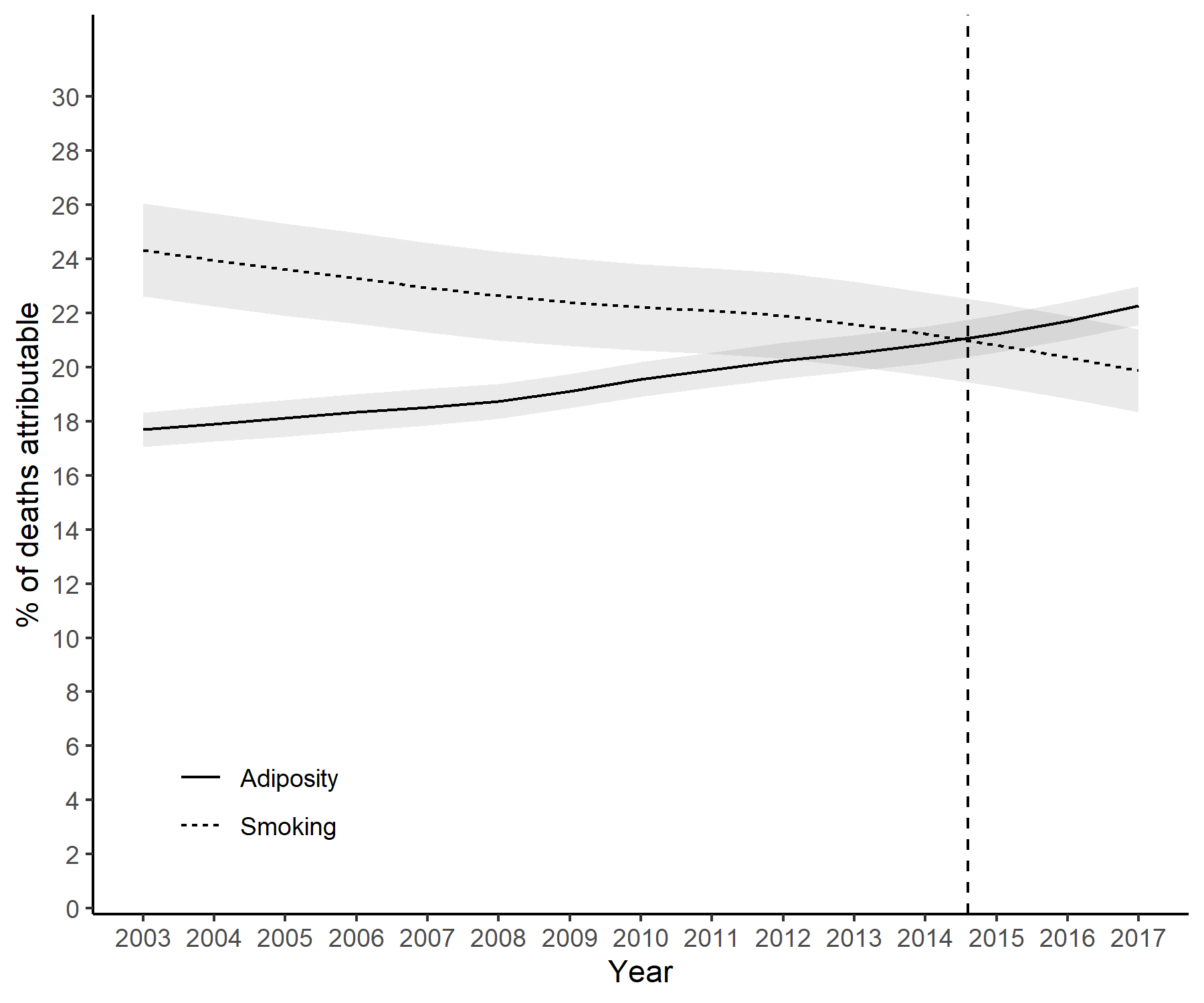


Shaded areas are 95% confidence bands. Vertical dashed line indicates cross-over.

Supplementary Figure 2. Percentage of all-cause deaths attributable to adiposity and smoking by sex using alternative RRs


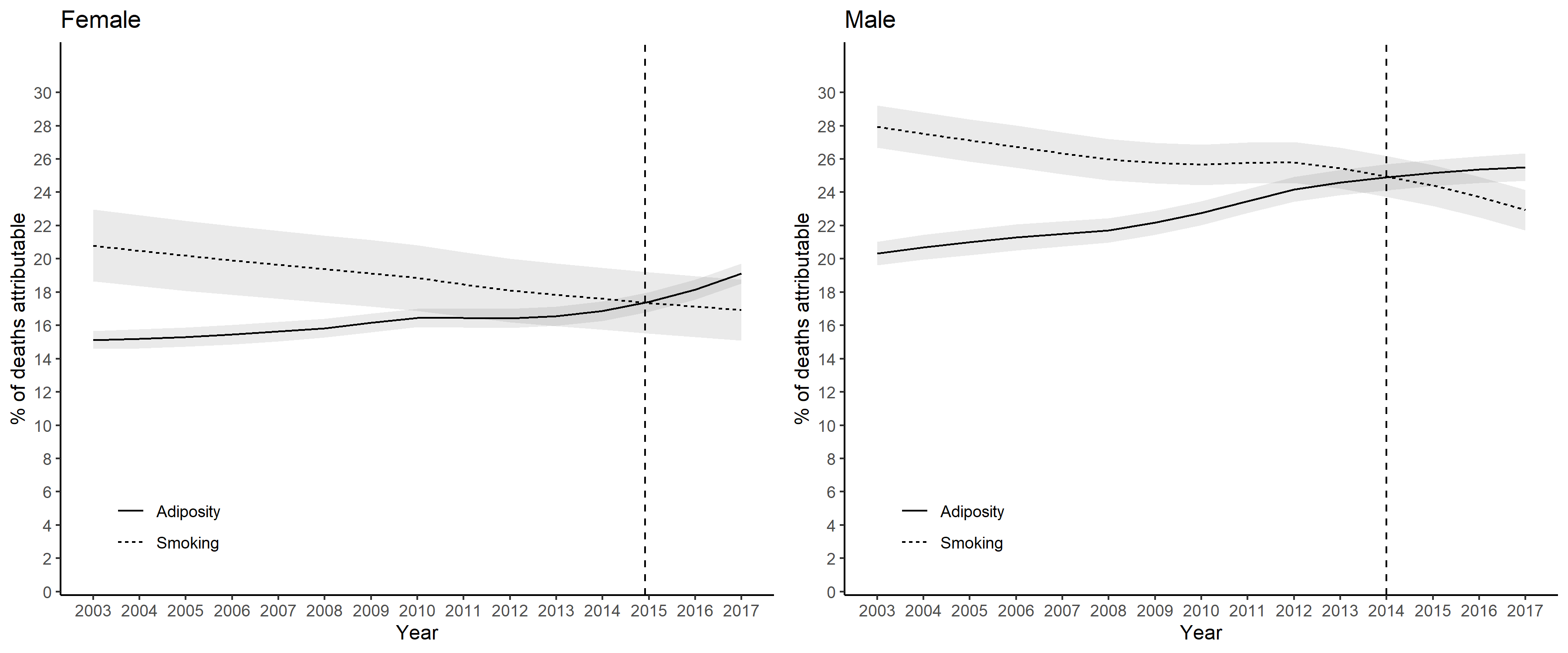


Shaded areas are 95% confidence bands. Vertical dashed line indicates cross-over.

Supplementary Figure 3. Percentage of all-cause deaths attributable to adiposity and smoking by age group using alternative RRs


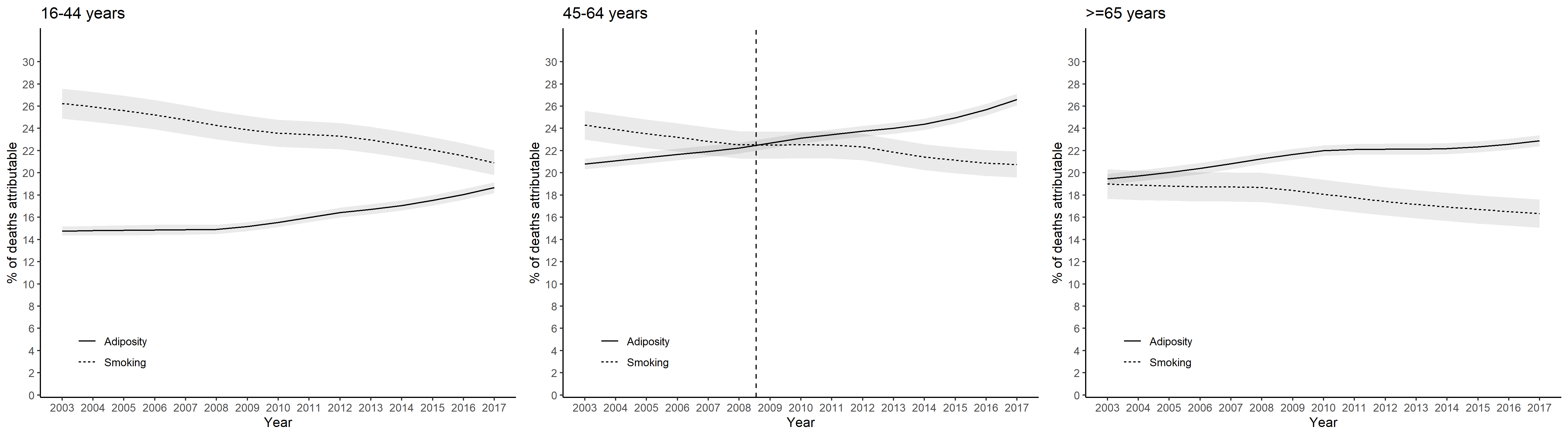


Shaded areas are 95% confidence bands. Vertical dashed line indicates cross-over.

Supplementary Figure 4. Percentage of all-cause deaths attributable to adiposity and smoking by age at completion of full-time education using alternative relative risks


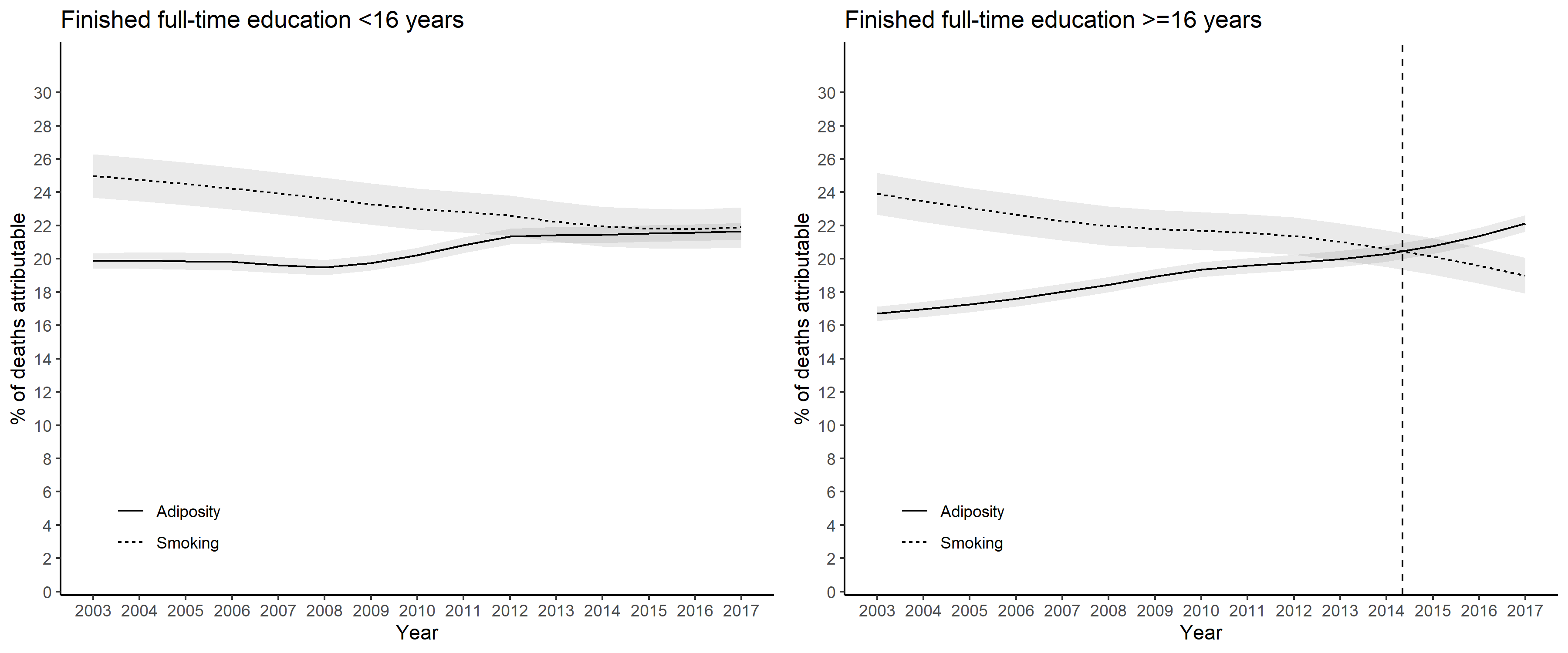


Shaded areas are 95% confidence bands. Vertical dashed line indicates cross-over.

**Data that was used in this study**

***Health Survey for England***

National Centre for Social Research, University College London. Department of Epidemiology and Public Health. (2010). *Health Survey for England, 2003*. [data collection]. *2nd Edition.*UK Data Service. SN: 5098, <http://doi.org/10.5255/UKDA-SN-5098-1>

National Centre for Social Research, University College London. Department of Epidemiology and Public Health. (2010). *Health Survey for England, 2004*. [data collection]. *2nd Edition.*UK Data Service. SN: 5439, <http://doi.org/10.5255/UKDA-SN-5439-1>

National Centre for Social Research, University College London. Department of Epidemiology and Public Health. (2011). *Health Survey for England, 2005*. [data collection]. *3rd Edition.*UK Data Service. SN: 5675, <http://doi.org/10.5255/UKDA-SN-5675-1>

National Centre for Social Research, University College London. Department of Epidemiology and Public Health. (2011). *Health Survey for England, 2006*. [data collection]. *4th Edition.*UK Data Service. SN: 5809, <http://doi.org/10.5255/UKDA-SN-5809-1>

National Centre for Social Research, University College London. Department of Epidemiology and Public Health. (2010). *Health Survey for England, 2007*. [data collection]. *2nd Edition.*UK Data Service. SN: 6112, <http://doi.org/10.5255/UKDA-SN-6112-1>

National Centre for Social Research, University College London. Department of Epidemiology and Public Health. (2013). *Health Survey for England, 2008*. [data collection]. *4th Edition.*UK Data Service. SN: 6397, <http://doi.org/10.5255/UKDA-SN-6397-2>

National Centre for Social Research, University College London. Department of Epidemiology and Public Health. (2015). *Health Survey for England, 2009*. [data collection]. *3rd Edition.*UK Data Service. SN: 6732, <http://doi.org/10.5255/UKDA-SN-6732-2>

NatCen Social Research, Royal Free and University College Medical School. Department of Epidemiology and Public Health. (2015). *Health Survey for England, 2010*. [data collection]. *3rd Edition.*UK Data Service. SN: 6986, <http://doi.org/10.5255/UKDA-SN-6986-3>

NatCen Social Research, University College London. Department of Epidemiology and Public Health. (2013). *Health Survey for England, 2011*. [data collection]. UK Data Service. SN: 7260, <http://doi.org/10.5255/UKDA-SN-7260-1>

NatCen Social Research, University College London. Department of Epidemiology and Public Health. (2014). *Health Survey for England, 2012*. [data collection]. UK Data Service. SN: 7480, <http://doi.org/10.5255/UKDA-SN-7480-1>

NatCen Social Research, University College London. Department of Epidemiology and Public Health. (2015). *Health Survey for England, 2013*. [data collection]. UK Data Service. SN: 7649, <http://doi.org/10.5255/UKDA-SN-7649-1>

NatCen Social Research, University College London. Department of Epidemiology and Public Health. (2018). *Health Survey for England, 2014*. [data collection]. *3rd Edition.*UK Data Service. SN: 7919, <http://doi.org/10.5255/UKDA-SN-7919-3>

NatCen Social Research, University College London, Department of Epidemiology and Public Health. (2019). *Health Survey for England, 2015*. [data collection]. *2nd Edition.*UK Data Service. SN: 8280, <http://doi.org/10.5255/UKDA-SN-8280-2>

NatCen Social Research, University College London, Department of Epidemiology and Public Health. (2019). *Health Survey for England, 2016*. [data collection]. *3rd Edition.*UK Data Service. SN: 8334, <http://doi.org/10.5255/UKDA-SN-8334-3>

University College London, Department of Epidemiology and Public Health, National Centre for Social Research (NatCen). (2020). *Health Survey for England, 2017*. [data collection]. *2nd Edition.*UK Data Service. SN: 8488, <http://doi.org/10.5255/UKDA-SN-8488-2>

National Centre for Social Research (NatCen), University College London, Department of Epidemiology and Public Health. (2020). *Health Survey for England, 2018*. [data collection]. UK Data Service. SN: 8649, <http://doi.org/10.5255/UKDA-SN-8649-1>

***Scottish Health Surveys***

Joint Health Surveys Unit, University College London. (2018). *Scottish Health Survey, 2003*. [data collection]. *4th Edition.*UK Data Service. SN: 5318, <http://doi.org/10.5255/UKDA-SN-5318-3>

Scottish Centre for Social Research and University College London. Department of Epidemiology and Public Health, *Scottish Health Survey, 2008* [computer file]. *3rd Edition.* Colchester, Essex: UK Data Archive [distributor], January 2016. SN: 6383, <http://dx.doi.org/10.5255/UKDA-SN-6383-3>

Scottish Centre for Social Research and University College London. Department of Epidemiology and Public Health, *Scottish Health Survey, 2009* [computer file]. *5th Edition.* Colchester, Essex: UK Data Archive [distributor], January 2016. SN: 6713, <http://dx.doi.org/10.5255/UKDA-SN-6713-3>

ScotCen Social Research and University College London. Department of Epidemiology and Public Health, *Scottish Health Survey, 2010* [computer file]. *3rd Edition.* Colchester, Essex: UK Data Archive [distributor], July 2016. SN: 6987, <http://dx.doi.org/10.5255/UKDA-SN-6987-3>

ScotCen Social Research, University College London. Department of Epidemiology and Public Health and University of Glasgow. MRC/CSO Social and Public Health Sciences Unit, *Scottish Health Survey, 2011* [computer file]. *4th Edition.* Colchester, Essex: UK Data Archive [distributor], January 2016. SN: 7247, <http://dx.doi.org/10.5255/UKDA-SN-7247-4>

ScotCen Social Research, University of Glasgow, MRC/CSO Social and Public Health Sciences Unit, University College London, Department of Epidemiology and Public Health. (2020). *Scottish Health Survey, 2012*. [data collection]. *4th Edition.*UK Data Service. SN: 7417, <http://doi.org/10.5255/UKDA-SN-7417-4>

ScotCen Social Research. (2020). *Scottish Health Survey, 2013*. [data collection]. *5th Edition.*UK Data Service. SN: 7594, <http://doi.org/10.5255/UKDA-SN-7594-5>

ScotCen Social Research. (2020). *Scottish Health Survey, 2014*. [data collection]. *4th Edition.*UK Data Service. SN: 7851, <http://doi.org/10.5255/UKDA-SN-7851-4>

ScotCen Social Research, *Scottish Health Survey, 2015* [computer file]. Colchester, Essex: UK Data Archive [distributor], December 2016. SN: 8100, <http://dx.doi.org/10.5255/UKDA-SN-8100-1>

ScotCen Social Research. (2017). *Scottish Health Survey, 2016*. [data collection]. UK Data Service. SN: 8290, <http://doi.org/10.5255/UKDA-SN-8290-1>

ScotCen Social Research. (2019). *Scottish Health Survey, 2017*. [data collection]. UK Data Service. SN: 8398, <http://doi.org/10.5255/UKDA-SN-8398-1>

ScotCen Social Research. (2020). *Scottish Health Survey, 2018*. [data collection]. *3rd Edition.*UK Data Service. SN: 8569, <http://doi.org/10.5255/UKDA-SN-8569-3>
